# Low-grade cribriform cystadenocarcinoma of salivary glands: report of two cases and review of the literature

**DOI:** 10.1186/1746-1596-8-28

**Published:** 2013-02-18

**Authors:** Liang Wang, Yang Liu, Xuyong Lin, Di Zhang, Qingchang Li, Xueshan Qiu, En-Hua Wang

**Affiliations:** 1 Department of Pathology, the First Affiliated Hospital and College of Basic Medical Sciences, China Medical University, Shenyang 110001, China; 2 Institute of Pathology and Pathophysiology, China Medical University, Shenyang 110001, China

**Keywords:** Salivary, Low-grade, Duct, Carcinoma, Cribriform, Cystadenocarcinoma

## Abstract

**Abstract:**

Low-grade cribriform cystadenocarcinoma (LGCCC) is a recently described rare tumor of salivary gland which exhibits clinically indolent behavior. This tumor predominantly consists of intraductal components and frequently exhibits papillary-cystic or cribriform proliferation pattern. Considering the histological features of LGCCC, it should be distinguished with papillocystic variant of acinic cell carcinoma, conventional salivary duct carcinoma, cystadenocarcinoma, polymorphous low-grade adenocarcinoma, carcinoma ex pleomorphic adenoma and mammary analogue secretory carcinoma. Herein, we presented two cases of LGCCC. One arose in the left parotid region in a 48-year-old male, and the other one arose in the right parotid gland in a 59-year-old female. For both cases, immunohistochemically, the luminal tumor cells showed diffuse expression of CK and S100; p63 and smooth muscle actin displayed a continuous rim of myoepithelial cells around all tumor islets; no myoepithelial cells were admixed with the luminal cells. Both patients were alive with no tumor recurrence or metastasis at follow-up.

**Virtual Slides:**

The virtual slide(s) for this article can be found here: http://www.diagnosticpathology.diagnomx.eu/vs/2593621568999135

## Background

Low-grade cribriform cystadenocarcinoma (LGCCC) is a rare neoplasm of salivary gland. Originally, it was described as a variant of salivary duct carcinoma (SDC) by Delgado et al. in 1996 [1]. LGCCC usually occurs in elder people with a female predominance of 2:1. Parotid gland is the most common site of involvement [1-6]. Presentation in the palate [7], submandibular gland [2], intraparotid lymph node [2,6] and accessory parotid gland [8] may occur but rare. LGCCC is characterized by the papillary-cystic or cribriform proliferation pattern and resembles the low-grade ductal carcinoma in situ or atypical ductal hyperplasia of the breast in histology and biological features. LGCCC was originally denominated as low-grade salivary duct carcinoma (LGSDC) in order to distinguish with the conventional SDC. In contrast with the LGCCC, conventional SDC exhibits highly aggressive malignancy and high-grade histology similar to an invasive ductal carcinoma of the breast. However, no definite association was found between LGCCC and conventional SDC; therefore, the third WHO classification regards this neoplasm as a variant of cystadenocarcinoma due to its cystic morphology [9].

Histologically, LGCCC was composed of single or multiple cystically enlarged ducts accompanied by adjacent intraductal proliferation. Various structures, such as cystic structures, loose cribriform and micropapillae pattern or solid area, could be observed in LGCCC. The typical cyst structures are lined by small or multilayered mild ductal cells with finely dispersed chromatin and small nucleoli. These tumor cells are diffusely strong positive for S100. Myoepithelial markers highlight the tumor cells rimming the cystic spaces, confirming the intraductal nature of LGCCC. Based on the histological features, LGCCC should be distinguished with other common parotid tumors including papillocystic variant of acinic cell carcinoma (PCV-ACC), conventional SDC, cystadenocarcinoma, polymorphous low-grade adenocarcinoma (PLGA), carcinoma ex pleomorphic adenoma and mammary analogue secretory carcinoma (MASC).

## Case presentation

### Clinical history

#### Case 1

A 48-year-old male was admitted to the First Affiliated Hospital of China Medical University in August of 2011 for further examination because of the mass in the left parotid region. The mass was soft, non-tender and did not adhere to the skin. Ultrasound examination revealed a nonhomogenous hypoechogenic mass with anechogenic areas measuring 21×12 mm. Examination by fine needle aspiration cytology was not performed. The patient underwent parotidectomy without radiotherapy. The patient was alive with no tumor recurrence or metastasis at 16 months of follow-up.

#### Gross features

The surgical specimen measured 3.5 cm in the greatest diameter. On cut surface, it showed a nonencapsulated tumor measured 2×1 cm. The tumor was whitish in color.

#### Microscopic features

**Figure 1:**
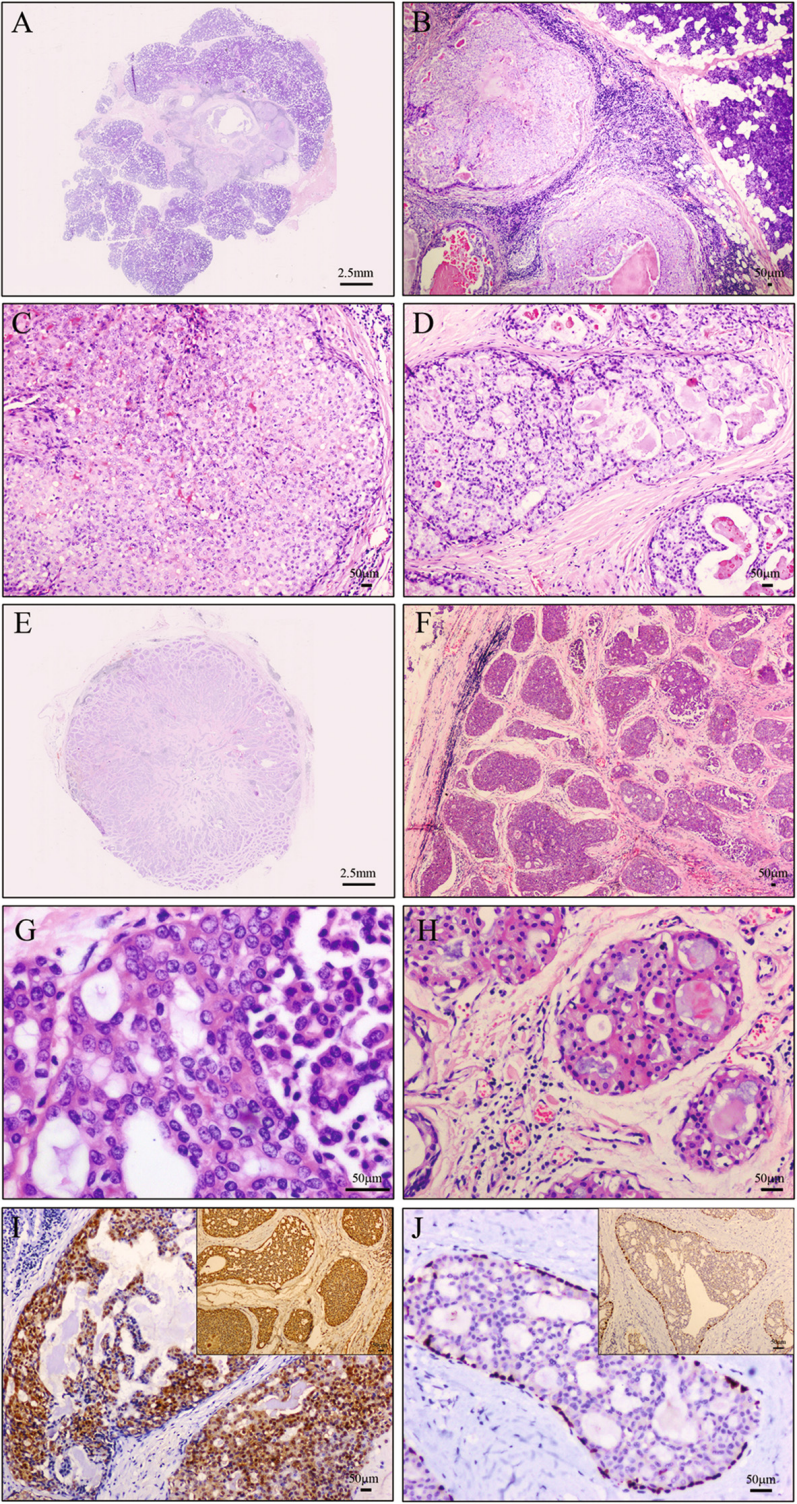
**Histological features and immunohistochemical staining of both cases. A**-**B**: Case 1, the tumor was demarcated from the surrounding slightly lipomatous parotid glands with a relative boundary. **C**-**D**: Case 1, within the tumor, solid and micropapillary pattern were observed. **E**-**F**: Case 2, the tumor was demarcated from the surrounding parotid glands with a clear boundary. **G**: Case 2, the tumor cells were uniform without significant cytologic and nuclear atypia. They displayed round to oval nuclei with fine chromatin and pale to amphophilic cytoplasm. **H**: Case 2, Apocrine differentiation of tumor cells was observed focally. **I**: The luminal tumor cells showed diffusive expression of S100 (Case 1 and 2). **J**: Immunostaining for p63 displayed a continuous rim of myoepithelial cells around all tumor islets (Case 1 and 2).

The tumor was demarcated from the surrounding slightly lipomatous parotid glands with a relative boundary (Figure 1A and B). The lesion was dominated by a large cystic space with multiple small well demarcated tumor islets in the fibrous stroma close to the central cyst. The architecture of these islets varied from solid (Figure 1C) to cribriform and micropapillary (Figure 1D). Within the lumen, pink secretions could be observed, but no comedo necrosis was identified. The tumor cells were uniform without significant cytologic and nuclear atypia. They displayed round to oval nuclei with fine chromatin and prominent nucleoli and pale to amphophilic cytoplasm. Apocrine differentiation of tumor cells was not obvious. No atypical mitosis was observed.

#### Immunohistochemistry

Immunohistochemically, the luminal tumor cells showed diffuse expression of CK and S100 (Figure 1I). Immunostaining for p63 (Figure 1J) and SMA displayed a continuous rim of myoepithelial cells around all tumor islets. No myoepithelial cells were admixed with the luminal cells. Tumor cells were negative for CEA, AR, ER, PR, EGFR, and Her-2/neu oncoprotein. Ki67 index was less than 5%.

#### Case 2

A 59-year-old female was admitted to the First Affiliated Hospital of China Medical University in May of 2012 for further examination because of the mass in the right parotid region. The physical examination showed a solid mass in the right parotid with slight tender and the mass did not adhere to the skin. Ultrasound examination revealed a homogenous hypoechogenic mass measuring 35×21×22 mm. Examination by fine needle aspiration cytology was not performed. The patient underwent parotidectomy without radiotherapy. The patient was alive with no tumor recurrence or metastasis at 7 months of follow-up.

#### Gross features

The surgical specimen measured 4 cm in the greatest diameter. On cut surface, it showed a well-circumscribed tumor measured 3×2 cm. The tumor was whitish in color.

#### Microscopic features

The tumor was demarcated from the surrounding parotid glands with a clear boundary (Figure 1E and F). The lesion was mainly composed of solid to cribriform and micropapillary structures, while the dilated cystic structures were rarely seen. No comedo necrosis was identified. The tumor cells were uniform without significant cytologic and nuclear atypia. They displayed round to oval nuclei with fine chromatin and pale to amphophilic cytoplasm (Figure 1G). Apocrine differentiation of tumor cells was observed locally (Figure 1H). No atypical mitosis was observed.

#### Immunohistochemistry

Immunohistochemically, the luminal tumor cells showed diffuse expression of CK, CK7 and S100 (Figure 1I). Immunostaining for p63 (Figure 1J) and smooth muscle actin (SMA) displayed a continuous rim of myoepithelial cells around all tumor islets. No myoepithelial cells were admixed with the luminal cells. Tumor cells were negative for CK20, AR, ER, PR, EGFR, and Her-2/neu oncoprotein. Ki67 index was less than 5%.

## Discussion

Like other rare tumors in parotid glands, LGCCC is a low-grade malignant tumor with indolent clinical behavior [10-12]. In the third WHO classification, it is listed under a separate heading although regarding as a variant of cystadenocarcinoma [9]. To date, there are nine papers totally reported 35 cases of LGCCC. The reported cases and the two cases in this paper were summarized in Table 1. In these patients, the majority of LGCCC occurred in older patients (median 62 years) without obvious gender preponderance (Male: Female=15:21; one case with gender unavailable) though the third WHO classification described that the tumor had a female predominance. Among the cases listed in Table 1, thirty-five tumors arose from parotid glands both in the superficial and deep lobes, including three cases occurred in the intraparotid lymph node and one occurred in accessory parotid gland. The remaining two tumors arose in the palate and submandibular gland. Therefore, all the LGCCCs reported by now occurred in large salivary glands except one controversial report on hard palate. The tumor size varied from 0.9 to 4 cm. Among the cases with follow-up available, no one had recurrence or died because of the tumor. According the clinically indolent behavior, most of the cases were treated with parotidectomy without radiotherapy.

**Table 1:** Reported cases of the low-grade cribriform cystadenocarcinoma in the English-Language Literature and their prognosis

**Case**	**Authors**	**Age**	**Sex**	**Location**	**Size**	**Treatment**	**Follow-up**
1	Delgado *et al*. 1996	58	M	Parotid (superficial lobe)	1 cm	Superficial parotidectomy	not mentioned
2		62	F	Parotid	0.7 cm	Parotidectomy	not mentioned
3		32	F	Right parotid (superficial lobe)	1.1 cm	Parotidectomy, radiotherapy	NED, 144 mos
4		63	M	Right parotid (superficial lobe)	1.3 cm	Parotidectomy	NED, 132 mos
5		74	M	Left parotid	1.8 cm	Parotidectomy	NED, 72 mos
6		56	F	Right parotid	1 cm	Parotidectomy	NED, 24 mos
7		42	M	Left parotid (superficial lobe)	1.2 cm	Parotidectomy	NED, 24 mos
8		69	F	Right parotid (intraparotid lymph node)	4 cm	Parotidectomy	NED, 24 mos
9		69	M	Left parotid	0.9 cm	Parotidectomy	-
10		52	F	Right parotid (deep lobe)	0.8 cm	Parotidectomy, radiotherapy	NED, 9 mos
11	Tatemoto *et al*. 1996	58	F	Palate	1 cm	Not mentioned	NED, 30 mos
12	Brandwein-Gensler *et al*. 2004	62	F 8 cases M 7 cases U 1 case	Parotid 15 cases (including one case from intraparotid lymph node)	Not mentioned	Not mentioned	NED, 12 mos
13		82					NED, 44 mos
14		78					NED, 17 mos
15		72					NED, 108 mos
16		93					NED, 24 mos
17							NED, 30 mos
18							NED, 62 mos
19		64		Submandibular gland 1 case			NED, 33 mos
20		66					Not mentioned
21		57					NED, 30 mos
22		63					Not mentioned
23		64					NED, 6 mos
24		62					NED, 132 mos
25		72					NED, 40 mos
26		76					NED, 24 mos
27		54					Not mentioned
28	Weinreb *et al*. 2006	50	F	Right parotid	2 cm	Superficial parotidectomy	NED, 5 mos
29		73	M	Left parotid	1.8 cm	Superficial parotidectomy and supraomohyoid neck dissection	NED, 60 mos
30		67	F	Right parotid	2.5 cm	Right total parotidectomy and chemotherapy and radiation therapy	Not mentioned
31	Arai *et al*. 2009	32	F	Right parotid	2.8 cm	Right partial parotidectomy	NED, 24 mos
32	Laco *et al*. 2010	50	F	Right parotideomasseteric area	1.5 cm	Enucleation of the tumor	NED, 24 mos
33	Nakatsuka *et al*. 2011	27	M	Accessory parotid gland	1.5 cm	Local excision of the tumor	NED, 3 mos
34		56	F	Left parotid	3 cm	Parotidectomy	NED, 12 mos
35	Weinreb *et al*. 2011	59	F	Left parotid (intraparotid lymph node)	3.5 cm	Not mentioned	Not mentioned
36	Current case	48	M	Left parotid	2cm	Parotidectomy	NED, 16 mos
37	Current case	59	F	Right parotid	3.5 cm	Parotidectomy	NED, 7 mos

*M *male; *F *female; *U *gender unavailable; *NED* no evidence of disease; *mos *months.

Grossly, the clinical suggestion of LGCCC is used to be Warthin tumor according to the morphologic feature, that is, a slowly growing cystic mass. Histologically, the tumor is unencapsulated and composed of single or multiple cysts with an intraductal proliferation. The cystic cavity is in line with cytologically bland ductal cells. The intraductal proliferation has a cribriform pattern with “sieve-like spaces” similar to breast proliferations. The majority of the tumor is intraductal; however, small areas of invasion may be present. A total of 7/37 (19%) cases summarized in Table 1 have focal stromal invasion. The tumor cells usually display little cytologic atypia and low mitotic rate [9]. Sometimes, the tumor cells have PAS-positive/diastase-resistant microvacuoles and yellow-brown, lipofuscin-like pigment. There is often associated hemorrhage, cholesterol clefts and hemosiderin-laden macrophages due to cyst rupture, while vascular and/or perineural invasion and comedonecrosis is absent in this tumor.

Immunohistochemically, LGCCC is characterized by diffusely strong positive for S100. However, Weinreb *et al*. [13] and Arai *et al*. [3] reported two cases of LGCCC with rarely negative for S100. All tumors examined for Her-2/neu oncoprotein, including our case, were negative. Most of the tumor structures have continuous myoepithelial rim confirmed by detection of myoepithelial markers, such as, CK5/6, CK14, SMA, and p63, thus clarifying an in situ nature of the neoplasm. Typically, admixture of luminal and myoepithelial cells is absent.

The differential diagnosis of LGCCC includes PCV-ACC, conventional SDC, cystadenocarcinoma, PLGA, carcinoma ex pleomorphic adenoma and MASC. PCV-ACC resembles LGCCC according to the intracytoplasmic PAS-positive/diastase-resistant granules (zymogen) and hemosiderin [14]. In contrast to LGCCC, the more common microcystic growth pattern in PCV-ACC is usually seen adjacent to cystopapillary areas and display granular basophilic cytoplasm. PCV-ACC does not exhibit predominance of the intraductal component in histology and the intracytoplasmic vacuoles are uniform in size. Compared with LGCCC, PCV-ACC is predominantly negative (about 90%) for S100 and focally expressed if present [9]. In addition, PCV-ACC mainly occurs in young people, while LGCCC usually affects older people.

Conventional SDC is a high-grade adenocarcinoma that is common in elder people over 50 years of age [9]. Histologically, SDC resembles a high-grade invasive ductal carcinoma of the breast, frequently accompanied by comedo necrosis and cribriform proliferation [9]. SDC exhibits an apocrine-like appearance with positivity for GCDFP-15 and androgen receptor, which is occasionally observed in LGCCC, but SDC is negative for S-100. In addition, SDC usually exhibits a high Ki-67 labeling index. Occasional cases of SDC in situ (SDCIS) have been described [15-17]. The in situ–like appearance or noninvasive cribriform growth pattern make it hard to distinguish between SDCIS and LGCCC. In contrast to LGCCC, the atypical cells in SDCIS exhibit high nuclear pleomorphism and AR-positive/S100-negative profile. Weinreb *et al*. [6] reported a series of 3 intraductal neoplasms interpreted as LGCCC with some nuclear atypia of the tumor cells, all expressing AR and in 2 cases also showing S100 expression. Thus, they consider their LGCCC cases to be a low-grade variant of SDC with a potential for transformation into a high-grade carcinoma. At present, the relationship between LGCCC and SDCIS remains unclear although the current WHO classification considering LGCCC as a variant of cystadenocarcinoma. LGCCC may be either a separate entity based on distinctive immunohistochemical profile or an extremely low-grade end of the spectrum of SDCIS.

Recently, a new entity in the salivary gland called MASC has been described [18,19], which shows a similar microvacuolar appearance to LGCCC and may have any of solid, cystic and papillary architectures. Similar to LGCCC, MASC is also positive for S100. All these characteristics make it difficult to distinguish between LGCCC and MASC. Although no cases of MASC with an intraductal growth pattern have been described, it should be noted that only one IDC/LGCCC was included in the control group tested for the ETV6-NTRK3 fusion in Skalova et al’s original description of MASC. For now, the presence of a complete myoepithelial layer around tumor nests is considered specific to LGCCC instead of MASC.

In the current WHO classification, LGCCC is regard as a variant of cystadenocarcinoma according to the histological features. However, conventional cystadenocarcinoma tends to be an invasive tumor and lacks the overall resemblance to intraductal breast proliferation. PLGA and carcinoma ex pleomorphic adenoma should also be considered when rendering a diagnosis of LGCCC. PLGA can be distinguished from LGCCC by its distinctive neurotropism and infiltrative lobular, trabecular, and tubular patterns. The transition from myoepithelium to stromal spindle cell and myxoid or cartilaginous stroma is the typical features of carcinoma ex pleomorphic adenoma, which is not observed in LGCCC.

Both of the cases exhibited the typical features of LGCCC, that is, intraductal proliferation and bland ductal cells arranged in a cribriform pattern whichresembled the low-grade breast lesions. Most of the tumor nests or cribriform structures in these two cases were rimming by the continuous myoepithelialium which confirmed by p63, thus, clarify an in situ nature of the neoplasm. The tumor cells in both cases were diffusely strong positive for S100. In addition, the Ki67 index in our cases was under 5%. All these findings supported the diagnosis of LGCCC, although the dilated cystic structures were not observed in Case 2.

## Conclusion

Our report illustrated two cases of LGCCC which was a rare salivary tumor. LGCCC should be considered in the differential diagnosis of salivary tumors to avoid rendering a wrong diagnosis of other highly aggressive malignant tumor of salivary gland.

## Consent

Written informed consent was obtained from the patient for publication of this case report and accompanying images. A copy of the written consent is available for review by the Editor-in Chief of this Journal.

## Competing interests

The authors declare that they have no competing interests.

## Authors’ contributions

LW analyzed the data and wrote the manuscript as a major contributor. YL, XL and DZ helped to perform the immunochemical staining. QL, XQ and EW helped to revise the discussion section of this manuscript. All authors have read and approved the final manuscript.
